# Crystal Gold

**Published:** 1876-11

**Authors:** Ambler Tees

**Affiliations:** Philadelphia, Pa.


### ARTICLE III.

### *Crystal Gold.*

BY AMBLER TEES, D. D. S., PHILADELPHIA, PA.

Read before the American Dental Convention.

In a letter recently addressed to Dr. A. J. Watts, the patentee and manufacturer of Watt's Crystal Gold, I made the following inquiries of him, which he very cheerfully answered:

1st. When was Crystal Gold first discovered?

2d. By whom?

3d. Under what circumstances?

4th. What is the difference between that now manufactured and the product of the first experimentation?

5th. What is the process of manufacturing at present?

6th. What is the best way to anneal, in case it should become damp?

During the winter of 1852-53, while Dr. Watts was lecturing in the western part of the State of New York on Chemistry and Electricity, particularly on the phenomena relating to Electro-Chemistry, he became acquainted with Dr. Ball, a dentist, who asked him whether it was possible to make Sponge Gold of a soft, porous and compressible nature. He was shown by Dr. Ball an article in a dental journal describing the process of making sponge gold by the well-known method of dissolving gold in Aqua Regia, precipitating by iron and gathering the resultant mass together after thorough washing by water. He informed Dr. Watts that Dr. Jackson, of Boston, had made and sold a lot of this; he had tried it but found it worthless.

After a few days deliberation Dr. Watts came to the conclusion that if gold in a very finely divided state, such as the precipitate just alluded to, was dissolved in mercury and the mercury afterwards dissolved out by means of nitric acid, the result would be a sponge. Acting upon this thought, on the 13th of January, 1853, he dissolved a five dollar gold piece in nitro-muriatic acid and proceeded as usual to obtain the pure pulverdlent gold in a dried condition and commenced his experiments. This was an unknown field to him, and consequently he was astonished and delighted at what followed. He dissolved about a pennyweight of the gold in five or six times its weight of mercury, and after thorough trituration, poured the mass into a small porcelain basin, and adding to it some dilute nitric acid, set it on the top of a hot stove and watched the rather slow process of the action of the acid upon the mercury. As the basin became hot, this became more active, and after a time ceased. He drew off the saturated acid and poured on a fresh supply of the strong undiluted acid, which soon removed all the remaining mercury, and revealed to his sight a beautiful mass of bright and shining crystals.

That was *Crystal Gold!* But how did it become crystallized? He found that if the temperature was kept down to or below 60° Fahrenheit during the whole period, from the first amalgamation to the first dissolving out of the mercury, the result was invariably an amorphous, brown spongy-looking mass, destitute of the least trace of crystallization when submitted to the microscope. So that his impromptu experiment had really at the outset the very conditions which secured the best results.

This form of gold was put upon the market, but the demand was for a more porous, lighter and softer article. After many experiments in Electrolysis, (decomposition by electricity) Mr. Watts at last succeeded in obtaining the beautiful formation which now comes to us. He thinks it best, always before using it, to anneal it on a sheet of platina foil, held over a broad alcohol flame until the foil becomes reddened.

Had it not been for the discovery of the welding properties of foil by Prof. Arthur, (see the April number of the *Dental News Letter*, 1855,) a short time after its introduction, I have no doubt that to-day Crystal Gold would have been the favorite gold with the best operators. The disposition on tbe part of the representative men in the profession at that time, seemed to be, to favor cohesive foil; and it soon effected a revolution in dentistry and its appliances. If it was possible for a dentist deceased only twenty years ago to visit the establishment of S. S. White in Philadelphia, he would hardly suppose himself to be in a dental depot.

It may not be uninteresting to you if I make a few extracts from the pens of two of the leading men of twenty years ago. In the October number of the *Dental News Letter*, 1854, Dr. Arthur writes:

"The new and very remarkable preparation of gold, lately introduced to the notice of the profession, under the name of sponge gold, crystallized gold, prepared gold, &c., goes farther than anything which has yet been offered to supply some of the defects of gold foil for our purpeses. It is unquestionably, in my estimation, an advance, and a great advance upon the ordinary foil as a material for filling teeth. I speak, of course, of the best specimens which have been produced. This gold cannot be called plastic in the strict sense of the term, for it cannot be moulded into a desired form. It simply possesses the property of firm cohesion, one part to another when subjected to pressure, and this goes far to make up for its lack of plasticity.

For the purpose in question, it is very nearly a complete substitute for it. It not only welds together in the mass when compressed, but if certain conditions are observed, one piece welds firmly to another, which has undergone compression. In this manner a cavity of any size may be filled, and the filling, under ordinary pressure, becomes a solid mass of pure gold. It becomes so dense, indeed, that it is not more readily cut than an ingot of pure gold. So advantageous are the properties it possesses for the important purpose in question, that if a firm hold can be found for any considerable portion of it, a filling may be completed to answer fully all the purposes of a filling without reference to the form of any other part of the cavity."

In an editorial in the same number, Dr. J. D. White says of it:

"This preparation of gold for filling teeth has had, we think, a pretty fair trial, at least in our hands. We are not very sanguine in our hopes of it ever meeting the high expectations of its inventors and more ardent advocates, although at first it looked more like the long sought for desideratum or substitute for gold foil, than anything else that we ever saw or tried. It is now in the 'collapsed stage' with us, and unless it receives some powerful and new stimulus from some new quarter it will most surely die. We made an honest endeavor to learn as much about its use as we could from its friends, and by experimenting with it for about one year, have failed to make satisfactory operations with it. Whether we did not succeed in learning all about the proper method of using it we know not, and we are therefore open to further instructions. If we mistake not, we were told that it required less time and labor than gold foil—in this we have been sadly disappointed—and that it would take hold of a cavity much better than foil, or in other words, a cavity that would not take foil, could be safely plugged with this; we heard of it actually welding itself to the walls of a cavity; that upon breaking open a tooth that had been plugged with it, the gold would be found to have so united with the dentine as almost to defy its separation. This may be true in plugging a dry tooth out of the mouth by applying great pressure; but as to obtaining such results in the mouth, it is purely imaginative. We must confess we have, as well as some of our friends, increased our business by its use, because we have been obliged to do our work twice, where once would have answered as well."

During the past five or six years I have used many ounces of Crystal Gold in filling cavities of decay, in the approximal surfaces especially; and as the months and years roll by, I have been much encouraged in its use, since the fillings compare favorably with those of cohesive foil in every respect. It is so soft and yet so cohesive that with the instruments and appliances now in use, the average dentist should be able to obtain with it the most beautiful results. If my business has been increased by its use, it has not been, as was the case with Dr. White, on account of the necessity of replacing lost fillings, but rather because the fillings look well, please well, and stand well.

In explaining my mode of working Crystal Gold I shall speak of:

1st. *The preparation of the cavity.* In this part of the operation just as much care must be exercised as for the reception of cohesive foil. The retaining pits should be so drilled and located that throughout the process of packing, the gold may remain perfectly firm. In a larger or shallow approximal cavity it is my practice to open a communication into the articulating cavity, if there be one, and if not, I make one. By this means I get a firm anchorage and fill otherwise difficult cavities with ease.

2d. *The preparation of the gold.* To divide the lump as it comes to us into pieces of the proper size, I use a sharp razor, cutting it into slabs of different thicknesses, which I again divide by means of two sharp pointed instruments into large or small pieces as the size of the cavity may demand. By this means I avoid mutilating the crystals and can work it in the same manner as heavy foil. The idea of preparing in this way was suggested to me by the facility with which a small slab which sometimes comes in the box to make up the one eighth ounce, was torn into small pieces. I use No 2 in preference to the other numbers.

3d. *Instruments to be used.* In using Crystal Gold it is very important that the dentist should have the proper instruments for introducing and condensing it. I think a good selection may be made from Dr. Ellis's set for plastic gold. I prefer those with flat points. I have had made for myself a modification of some of these points, which I find well adapted for small and medium sized cavities. For filling large cavities I use No. 53 of Dr. Atkinson's points, to be found in S. S. White's catalogue, and a modification of it with finer serations.

4th. *Exclusion of moisture.* Crystal Gold will not tolerate moisture in any form. To insure a good filling, therefore the cavity must be well protected; and the rubber dam secured by the various shaped clamps, or the waxed ligatures, enable us to do this in a very satisfactory manner.

5th. *Annealing.* If the gold works well and to my satisfaction, it is not my practice to anneal it. If it shows any indication of being damp, as I pick up each piece with the point of the plugger, I hold it for a few seconds about an inch above a very small flame of a spirit lamp.

6th. *The Electro-Magnetic Plugger.* This ingenious piece of mechanism is certainly a very valuable auxillary in the use of Crystal Gold, especially in large cavities. The blows are more uniform, if not more effective than those given by the hand mallet, and on account of their rapidity seem to be more grateful to the patient. One great objection to it is the noise, which tends to make both patient and operator nervous; and by many is almost unendurable. Let us hope that the inventive talent of some member of our profession may speedily remove this obejction.—*Penna. Journal of Dental Science.*

